# Reproductive and Developmental Toxicity of Environmental Chemicals: From Mechanisms to Population Evidence

**DOI:** 10.3390/toxics14070598

**Published:** 2026-07-09

**Authors:** Jie Zhang, Jisheng Nie, Tao Chen

**Affiliations:** 1 School of Public Health, Suzhou Medical College of Soochow University, Suzhou 215123, China; zhangjie_78@suda.edu.cn; 2 School of Public Health, Shanxi Medical University, Taiyuan 030001, China; niejisheng@sxmu.edu.cn; 3 Jiangsu Key Laboratory of Preventive and Translational Medicine for Major Chronic Non-Communicable Diseases, Suzhou 215123, China

## 1. Introduction

Environmental pollution has become a global public health crisis, posing profound threats to human reproductive stability and fetal developmental homeostasis [1]. The developmental window spanning from gestation to early postnatal life represents the most susceptible stage to exogenous environmental stressors. Consistent with the Developmental Origins of Health and Disease (DOHaD) theory, early-life environmental toxicant exposure induces persistent biological alterations via endocrine disruption, oxidative stress, inflammation, and epigenetic reprogramming, thereby exerting lifelong adverse impacts on human health [2]. Despite accumulating evidence confirming the reproductive and developmental toxicity of environmental factors, critical research gaps remain in mechanistic understanding, real-world mixture risk assessment, and actionable intervention strategies. This Special Issue, titled “Reproductive and Developmental Toxicity of Environmental Factors”, integrates cutting-edge research across chemical toxicology, multi-omics mechanistic research, population-based human epidemiological investigation, and pathological mechanism review. Here, we summarize the core findings of published studies and propose future research directions for the field.

## 2. Overview of Published Articles

Heavy metals remain among the most extensively studied environmental toxicants in reproductive and developmental research. Cadmium, a typical environmental heavy metal, exerts specific deleterious effects on adolescent reproductive neurodevelopment. Arteaga-Silva et al. (contribution 1) demonstrated that cadmium exposure disrupted the hypothalamic kisspeptin–GnRH axis in male pubertal rats, downregulated key gene expression at different developmental stages, reduced serum testosterone and antioxidant capacity, and ultimately delayed sexual maturity. These findings confirm cadmium’s dual role as a neurotoxicant and endocrine disruptor during critical reproductive developmental windows. Moving beyond single-metal toxicity, combined heavy metal exposure represents a more realistic environmental scenario. Sun et al. (contribution 2) verified that combined exposure to lead, cadmium, manganese, and arsenic could induce gestational diabetes mellitus (GDM)-like metabolic dysfunction in pregnant rats, including elevated blood glucose, insulin resistance, and hepatic pathological damage. Mechanistically, disrupted glycerophospholipid metabolism and altered *Insig1* expression were identified as core regulatory pathways, revealing the molecular basis of heavy metal-induced gestational metabolic injury and highlighting the urgency of evaluating metal mixtures in environmental risk assessment.

Daily chemical disinfectants and industrial raw materials warrant rigorous toxicological scrutiny due to widespread human exposure. Melin and Hrubec (contribution 3) focused on quaternary ammonium compounds (QACs), specifically alkyl dimethyl benzyl ammonium chloride (ADBAC) and didecyl dimethyl ammonium chloride (DDAC), which are ubiquitous in domestic and industrial disinfectants. Their study confirmed that both direct and in utero exposure to these agents causes persistent declines in sperm concentration and motility in F0 and F1 male mice. Notably, epigenetic modifications of chromatin regulatory enzymes in testis tissue mediated this multi-generational reproductive toxicity. However, no trans-generational inheritance was observed in the F2 generation, clarifying the unique epigenetic inheritance patterns of QAC-induced reproductive damage. Regarding industrial chemical safety, Eom et al. (contribution 4) conducted repeated-dose and reproductive/developmental toxicity testing of calcium nitrate tetrahydrate based on OECD standard protocols. The results demonstrated that high-dose exposure reduced F1 litter size and increased post-implantation loss. The study further clarified the differential no observed adverse effect level (NOAEL) values for male and female reproductive toxicity, filling critical toxicological data gaps for this widely used industrial raw material and supporting regulatory decision-making.

Prenatal pharmaceutical exposure represents another critical source of developmental disruption. In a pioneering human study, Liu et al. (contribution 5) innovatively identified that prenatal dexamethasone exposure mediates epigenetic modification of the low-density lipoprotein receptor (LDLR) via the glucocorticoid receptor (GR)/histone deacetylase 2 (HDAC2) axis, leading to persistent hypercholesterolemia in male offspring. Notably, this human study demonstrated that LDLR H3K27ac levels in peripheral blood mononuclear cells (PBMCs) were validated as a non-invasive early warning biomarker for fetal metabolic susceptibility, providing a novel, practical tool for prenatal developmental toxicity monitoring and human early-life metabolic risk prediction.

Among endocrine-disrupting chemicals (EDCs), bisphenol A (BPA) remains a research priority due to its ubiquitous environmental presence and well-documented health risks. Wu et al. (contribution 6) explored the neurobehavioral toxicity of BPA using a zebrafish model, finding that BPA exposure inhibits the expression of the circadian clock gene *nr1d1*, triggers neuroinflammation and dopamine system imbalance, and induces anxiety-like behaviors in zebrafish larvae. Additionally, from a chronotoxicology perspective, the study introduces a temporal dimension to EDC research, suggesting that circadian state may influence the evaluation of BPA toxicity and the assessment of intervention effects. Crucially, activating NR1D1 effectively counteracted the adverse effects, identifying a novel therapeutic target for BPA-induced neurodevelopmental toxicity and demonstrating the potential of mechanism-based interventions against EDC exposure.

Air pollution, a ubiquitous environmental exposure, is strongly linked to adverse reproductive and developmental outcomes, and high-quality human studies stand as the most authoritative evidence to characterize such real-world health hazards. Dai et al. (contribution 7) conducted a retrospective case–control study in Wuhan, China, confirming the trimester-specific adverse effects of air pollutants on gestational diabetes mellitus (GDM). Nitrogen dioxide (NO_2_) exposure increased GDM risk throughout pregnancy, whereas fine particulate matter (PM_2.5_) and sulfur dioxide (SO_2_) exerted toxic effects specifically during early and mid-pregnancy. These human study-derived findings provide targeted environmental protection recommendations for pregnant women at different gestational stages, emphasizing the importance of temporal specificity in exposure assessment. Complementing this work, Zhang et al. (contribution 8) carried out a nationwide cross-sectional human population survey focusing on persistent air pollution waves, revealing that long-duration PM_10_ wave exposure significantly impairs male sexual function. Furthermore, depression mediates the adverse correlation between particulate pollution and sexual health, highlighting the dual physical and psychological reproductive damage of air pollution, with particular vulnerability among young and low-risk human populations. Collectively, these two independent human studies deliver population-level evidence that directly guides public health interventions to mitigate air pollution-related reproductive harms in real human communities.

Finally, two systematic review articles provided comprehensive mechanistic frameworks for understanding environmental toxicant action. Wang et al. (contribution 9) systematically elaborated the disruption mechanism of common environmental hazards on the epididymal immune microenvironment, revealing the core role of immune homeostasis imbalance in male infertility. Beltran-Castillo et al. (contribution 10) reviewed the adverse effects of prenatal tobacco smoke and vaping aerosol exposure on fetal white matter development. Together, these reviews demonstrate how environmental chemicals compromise male reproductive tract immunity and fetal neural development.

## 3. Conclusions and Future Perspectives

Collectively, the studies in this Special Issue demonstrate that heavy metals, daily disinfectants, industrial chemicals, exogenous hormones, endocrine disruptors, and air pollutants compromise human reproductive health and fetal development through endocrine disruption, neuroinflammation, epigenetic reprogramming, and metabolic perturbation. These findings underscore the profound vulnerability of early developmental windows to even low-dose environmental exposures and their lifelong health consequences.

Despite significant advancements, critical knowledge gaps remain. Real-world human exposure involves complex pollutant mixtures rather than isolated substances, yet most current studies remain confined to single toxicants [3]. Future research should prioritize elucidating the synergistic or antagonistic mechanisms of combined exposures and establish comprehensive risk assessment frameworks for mixed pollutants, utilizing multi-omics approaches. Equally urgent are protective interventions. Research efforts must extend beyond mechanistic elucidation to rigorously evaluate the efficacy of dietary antioxidants, targeted pharmacological strategies, and practical exposure reduction strategies aimed at mitigating reproductive and developmental toxicity. Additionally, emerging contaminants, including microplastics and novel endocrine-disrupting chemicals, require thorough evaluation, preferably employing human-relevant models such as organoid systems integrated with artificial intelligence methodologies to address the limitations inherent in conventional animal toxicology [4]. Ultimately, large-scale multi-center prospective cohort studies are essential to validate long-term developmental risks and to guide the formulation of evidence-based health protection policies for vulnerable populations.

## Data Availability

The original contributions presented in this study are included in the article. Further inquiries can be directed to the corresponding author.
